# Organic Acid to Nitrile: A Chemoenzymatic Three‐Step Route

**DOI:** 10.1002/adsc.202201053

**Published:** 2022-12-28

**Authors:** Margit Winkler, Melissa Horvat, Astrid Schiefer, Victoria Weilch, Florian Rudroff, Miroslav Pátek, Ludmila Martínková

**Affiliations:** ^1^ Institute of Molecular Biotechnology Graz University of Technology Petersgasse 14 A-8010 Graz Austria; ^2^ acib GmbH Krenngasse 37 8010 Graz Austria; ^3^ Institute of Applied Synthetic Chemistry TU Wien Getreidemarkt 9/OC-163 A-1060 Vienna Austria; ^4^ Institute of Microbiology of the Czech Academy of Sciences Vídeňská 1083 CZ-142 20 Prague Czech Republic

**Keywords:** chemoenzymatic synthesis, carboxylic acid reductase (CAR), aldoxime dehydratase (Oxd), cyanide-free nitrile synthesis, nitriles, oxidoreductases

## Abstract

Various widely applied compounds contain cyano‐groups, and this functional group serves as a chemical handle for a whole range of different reactions. We report a cyanide free chemoenzymatic cascade for nitrile synthesis. The reaction pathway starts with a reduction of carboxylic acid to aldehyde by carboxylate reductase enzymes (CARs) applied as living cell biocatalysts. The second – chemical – step includes *in situ* oxime formation with hydroxylamine. The final direct step from oxime to nitrile is catalyzed by aldoxime dehydratases (Oxds). With compatible combinations of a CAR and an Oxd, applied in one‐pot two‐step reactions, several aliphatic and aryl‐aliphatic target nitriles were obtained in more than 80% conversion. Phenylacetonitrile, for example, was prepared in 78% isolated yield. This chemoenzymatic route does not require cyanide salts, toxic metals, or undesired oxidants in contrast to entirely chemical procedures.

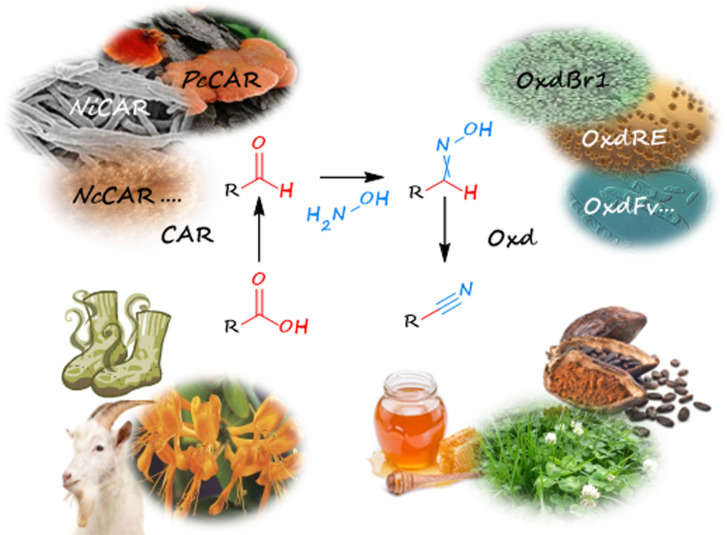

## Introduction

Cyano‐groups are present in various bioactive compounds, pharmaceuticals, agrochemicals, dyes, electronic materials and, in addition, they serve as a chemical multi‐tool for a whole range of different reactions.^[1]^ Cyano‐groups are precursors for primary and secondary amides, amidines, imidines, aldehydes, ketones, carboxylic acids, amines,^[2]^ nitrile‐oxides, nitrile imines and *N*‐heterocyclic compounds,^[3]^ cyanamides, thiocyanates and cyanate esters.^[4]^ As a general trend, nitriles evoke similar odour perceptions as their corresponding aldehydes. Due to their much greater stability, they were employed in products such as soaps, detergents, cleaners and personal care products.^[5]^ Phenylacetonitrile, 2‐methylbutyronitrile and 3‐methylbutyronitrile, for example, are natural, volatile, bioactive compounds. Their biosynthesis from amino acids *via* aldoximes is triggered by herbivore attack in plants like *Populus trichocarpa* ^[6]^ and *Fallopia sachalinensis*.^[7]^ Nevertheless, nitriles are synthesized predominantly by addition or substitution of leaving groups by cyanide.^[4]^ Cyanide salts are highly toxic, and therefore, cyanide free routes to nitriles are emerging.^[8]^ The research groups of Asano and Gröger reported a number of cyanide‐free syntheses of (aryl‐)aliphatic nitriles by aldoxime dehydratases, including enantioselective reactions.^[9]^ Attempting to utilize biorenewable resources, Gröger and co‐workers started the reaction from aliphatic alcohols available by reducing the biobased fatty acids.^[10]^ The alcohols were oxidized to aldehydes by NaOCl in the presence of a nitroxyl‐radical catalyst, and the whole cascade from alcohol to nitrile was carried out without isolating the intermediates (aldehyde, oxime). In contrast, we have proposed to start the nitrile synthesis with carboxylic acids.

**Scheme 1:**
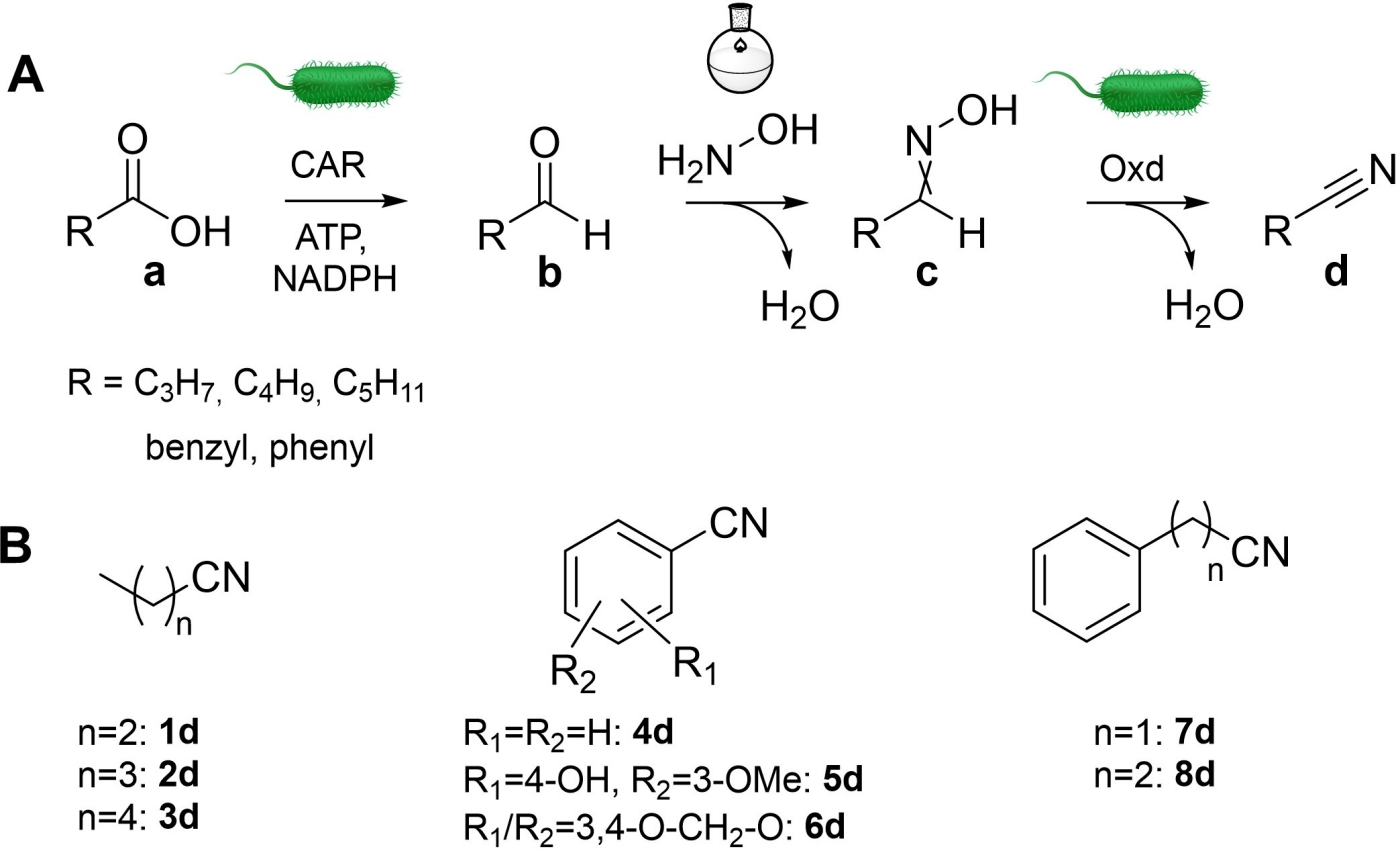
A Chemoenzymatic route from acid to nitrile. CAR**=**carboxylic acid reductase, Oxd**=**aldoxime dehydratase; B: Nitrile target compounds.

We recently communicated the first results on a chemoenzymatic cascade from carboxylic acids to nitriles that involves the enzymatic reduction of the acid to the aldehyde mediated by a carboxylic acid reductase from *Neurospora crassa* (*Nc*CAR),^[11]^ chemical oxime formation and finally the dehydration of oxime to nitrile catalyzed by an aldoxime dehydratase from *Bradyrhizobium sp*. (OxdBr1) (Scheme 1A).^[12]^ Carboxylic acids are activated by carboxylic acid reductases (CARs)^[13]^ at the expense of ATP, and reduced in form of an enzyme bound thioester by NADPH. Aldoxime dehydratases (Oxds) are heme‐proteins which operate independently of other co‐factors and remove a molecule of water from aldoximes.^[14]^

In this work, we sought to expand the scope of nitriles accessible through this cascade by exploring a larger toolbox of CARs and Oxds and extending the range of investigated substrates. Hence, representatives of aliphatic, aryl‐aliphatic and aromatic aldehydes were used for substrate screening. Selected substrates were used at preparative scale.

## Results and Discussion

**Figure 1:**
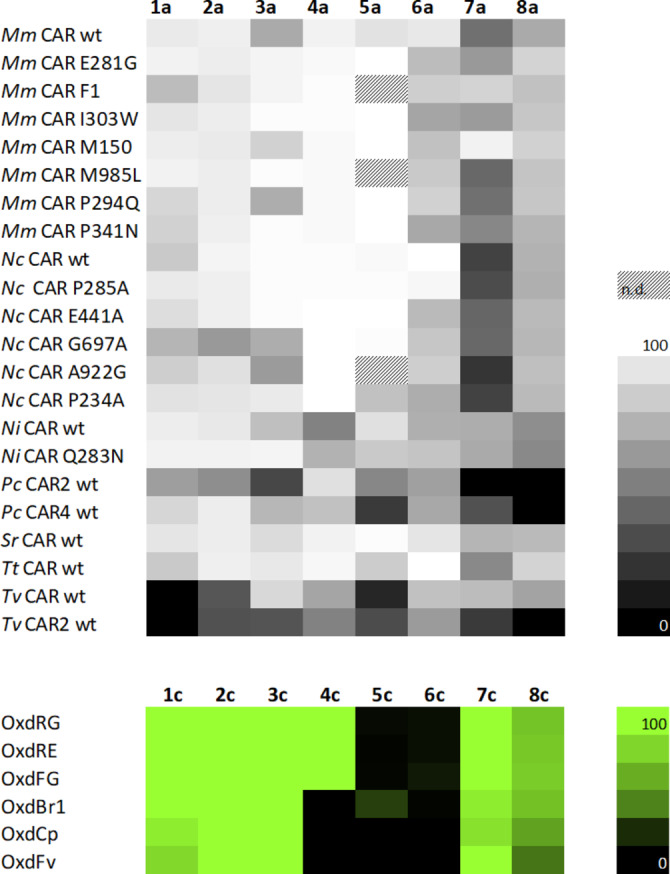
Top: Consumption of carboxylic acids in chemoenzymatic transformation of **a** (20 mM) to **c** in % with a panel of CAR enzymes in the presence of 1.5 equivalents of hydroxylamine. n.d.=not determined. Bottom: Conversion of **c** (10 mM) to **d** in % with Oxds in whole cells.

Carboxylic acid reductases are able to reduce a large variety of chemical structures including aliphatic, aryl‐aliphatic, aromatic and heterocyclic carboxylates with various substitutions.^[13]^ We selected a diverse variety of CARs to cover a possibly broad substrate scope. Type I CARs from *Mycobacterium marinum* (*Mm*CAR), *Nocardia iowensis* (*Ni*CAR), *Segniliparus rotundus* (*Sro*CAR), and promising variants from prior studies were included.[^15^, ^16^] Ascomycotal type III CARs from *Thermothelomyces thermophila* (*Tt*CAR) and a selection of promising variants from *Neurospora crassa* (*Nc*CAR)^[17]^ as well as basidiomycotal type IV CAR from *Pycnoporus cinnabarinus* (*Pc*CAR), and *Trametes versicolor* (*Tv*CAR) were also studied.^[18]^ These CARs were screened using the enzymes in form of a living cell biocatalyst in order to benefit from cellular metabolism for co‐factor supply.^[19]^ Hydroxylamine was added to those reactions to furnish *in situ* formation of oximes and at the same time to remove toxic aldehyde. Figure 1 (top) summarizes the consumption of carboxylic acids and gives an indication which CARs are most suitable for the reduction of the investigated substrates (Scheme 1B). Type I and type III CARs gave higher oxime yields on average than type IV CARs.

The product distribution of these chemoenzymatic transformations of **a** to **c** strongly depended on the chemical structure of the substrate. In case of aliphatic short chain acids **1 a**–**3 a**, for example, aldehyde **b** accumulated and did only partly react further to mixtures of *E* and *Z* oxime (Table 1, Supporting information Figure S1). On the contrary, oxime **5 c** formation was very efficient under the applied conditions in case of vanillin **5 b**, which is produced quantitatively with ten of the applied CAR enzymes from **5 a** (Figure S2). This and further data (not shown) show a strong impact of substrate structure on the oximation rate. Oxime formation is a two step process that starts with nitrogen base attack of the carbonyl to give an aminal that is dehydrated in the second step.^[20]^ At neutral pH, the first step is fast and acid‐catalyzed dehydration becomes the rate‐limiting step. The particular impacts of aldehyde‐hydrate equilibria, compound solubility and stereo‐electronic effects remains to be dissected. In our screening reactions, hydroxylamine was applied in 1.5 fold excess like in the work of Gröger *et al*.^[10]^ (Figure 1).

**Table 1:** Product distribution of selected chemoenzymatic transformations from acid **a** to oxime **c**.

Substrate	CAR	Residual **a** [%]^[a]^	Aldehyde **b** [%]	*E*/*Z* oxime **c** [%]^[b]^	Alcohol **e** [%]^[c]^
**1 a**	*Mm*CAR E281G	6	62	25	7
**2 a**	*Tt*CAR wt	8	30	56	6
**3 a**	*Nc*CAR wt	1	69	28	2
**4 a**	*Tt*CAR wt	4	18	77	1
**5 a**	*Mm*CAR E281G	0	0	80	20
**6 a**	*Tt*CAR wt	0	31	66	3
**7 a**	*Mm*CAR wt	10	14	76	0
**8 a**	*Mm*CAR E281G	11	10	49	20

^[a]^ unreacted **a**; ^[b]^ sum of *E* and *Z* oxime; ^[c]^ respective alcohols are a result of competing over‐reduction of the aldehyde.

**Figure 2:**
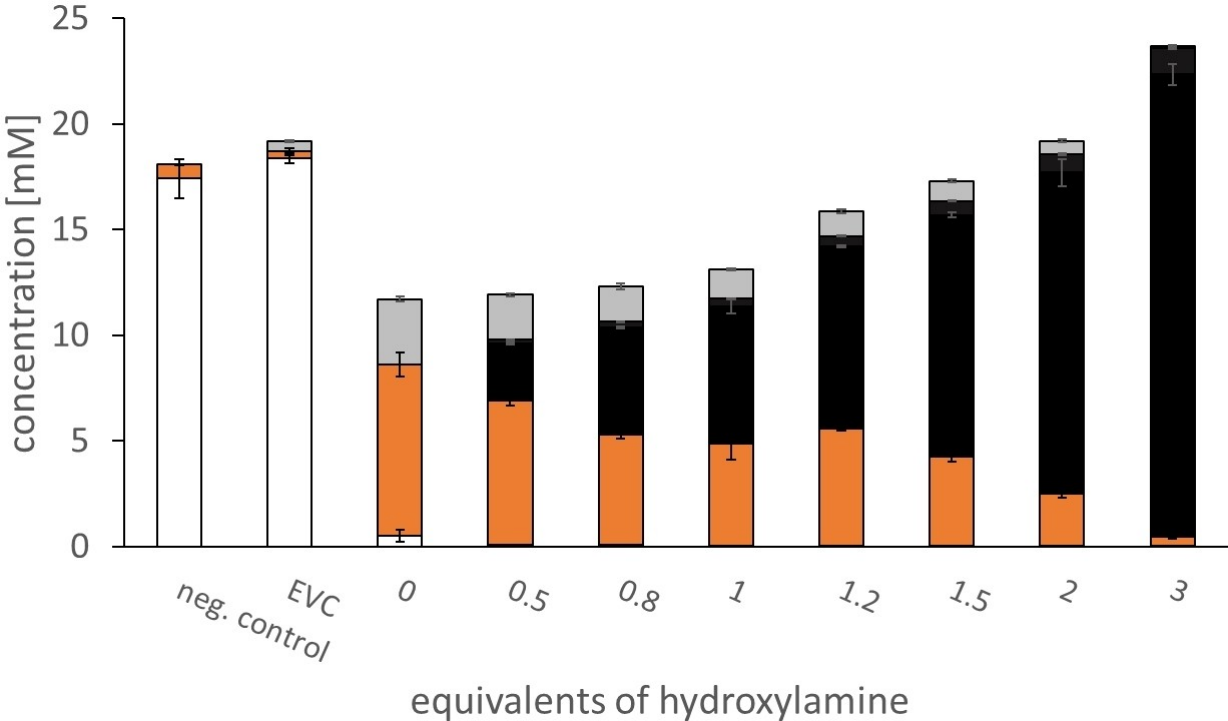
Effect of hydroxylamine concentration on oxime formation in *Nc*CAR mediated whole cell reductions of **4 a** (20 mM). Error bars are shown for technical triplicates. EVC: empty vector control. **4 a**: white; **4 b**: orange; *E*/*Z*‐**4 c**: black; **4 e**: grey.

We next tested different ratios of substrate to hydroxylamine, using **4 a** as a substrate for recombinant wild‐type *Nc*CAR. High concentrations of hydroxylamine (100 mM) were reported to lead to 12% inhibition of *Nc*CAR that had been purified from its natural host *Neurospora crassa*.^[21]^ Sub‐stoichiometric and stoichiometric amounts of hydroxylamine yielded less than 25% of oximes, significant amounts of undesired benzyl alcohol **4 e** and a poor recovery rate (Figure 2). Hydroxylamine in excess lead to a decreased over‐reduction of **4 b** to **4 e** and increased oxime yield. The more hydroxylamine was present, the less **4 b** accumulated, to the benefit of the mass balance and overall nitrile yield. Using three‐fold excess of hydroxylamine, **4 a** was near quantitatively converted to the desired *E*/*Z* mixture of oximes **4 c**. Inhibitory effects on *Nc*CAR were not encountered in this setup. However, competitive inhibition of Oxd by hydroxylamine was reported^[22]^ and we opted for 1.5 equivalents in most experiments. Ultimately, hydroxylamine concentration should be included in cascade optimization endeavours.

The aldoxime dehydratase step of the cascade was also tested in screening experiments first (Figure 1, bottom). Here, the oximes were incubated with various Oxds in form of whole cell biocatalysts. Full conversion to nitriles was observed for several enzyme/substrate combinations. Aliphatic aldoximes **1 c**–**3 c** and aryl‐aliphatic aldoxime **7 c** are excellent substrates for well‐studied OxdRG, OxdRE, OxdFG^[22]^ and OxdBr1.^[23]^ The new Oxds from the ascomycetous fungus *Fusarium vannetenii* strain 77‐13‐34 (OxdFv)^[24]^ and the actinobacterium *Corynebacterium pacaense* Marseille‐P2417 (OxdCp) are also suitable for the transformation of these oximes, with slightly lower conversions in some cases. All enzymes also transformed aryl‐aliphatic aldoxime **8 c**, albeit at lower conversions than its analog **7 c**. We observed full conversion of **4 c** by OxdRG, OxdRE, OxdFG, whereas OxdBr1 and the new Oxds did not accept this oxime as substrate. In contrast, of all tested Oxds only OxdBr1 transformed vanillin oxime **5 c** with 25% conversion. Piperonylic nitrile **6 d** was only produced in 10% conversion with OxdFG under the given conditions.

**Figure 3:**
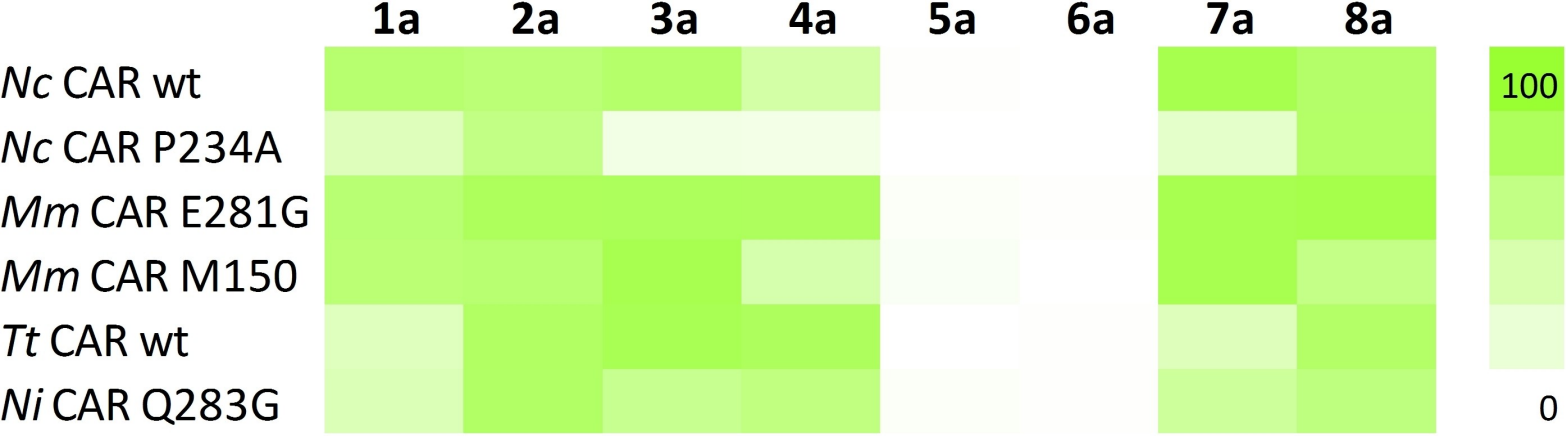
Nitrile formation in analytical scale sequential one‐pot cascades. Conversion of **a** (10 mM) to **d** in % with CARs and Oxds in whole cells at pH 7.5. OxdBr1 was used for **1 a**–**3 a** and **5 a**–**8 a**. OxdRG was used for **4 a**. Reaction time **1 a**–**3 a** and **7 a**–**8 a**: 1 h, Reaction time **4 a**–**6 a**: 16 h.

The best CAR variants were applied for analytical scale cascade reactions. OxdRG was used for substrate **4 a**, while the CARs were combined with OxdBr1 for all other substrates. Figure 3 shows the levels of detected nitriles. Aliphatic substrates **1 a**–**3 a** were fully consumed by wild‐type *Nc*CAR and the two *Mm*CAR variants. Corroborating the results observed before (Table 1), alcohol **e** was the most prominent side product (≤20%) and also minor amounts of **b** and **c** were found. In case of **4 a**, *Mm*CAR E281G and *Tt*CAR showed full substrate conversion, up to 9.3 mM of **4 d** and traces of residual **4 c** and **4 b**. Full conversion of **5 a** and **6 a** was achieved with at least two CARs, however, the major products were **5 b/5 c** and **6 b/6 c/6 e**, respectively, whereas desired **5 d** and **6 d** were only detected in low amounts. This result was not surprising, as inefficient oxime dehydration for this substrate class was observed already in the screening experiments (Figure 1). For aromatic oximes, the ideal Oxd yet needs to be discovered or customized for this purpose by protein engineering. By contrast, in case the oxime group is in a distance of at least one CH_2_ group from the phenyl moiety like in **7 a** and **8 a**, respectively, the chemoenzymatic cascade yields **7 d** or **8 d** as the major product at full conversion. In case of **8**, *Nc*CAR variants in combination with OxdBr1 yielded **8 c** as the major unreacted intermediate, while *Mm*CAR M150 produced predominantly **8 e**. *Mm*CAR M150 had been designed for alcohol formation^[16]^ and indeed gave on average higher levels of **e** than any other CAR variant in our experiments.

**Scheme 2:**
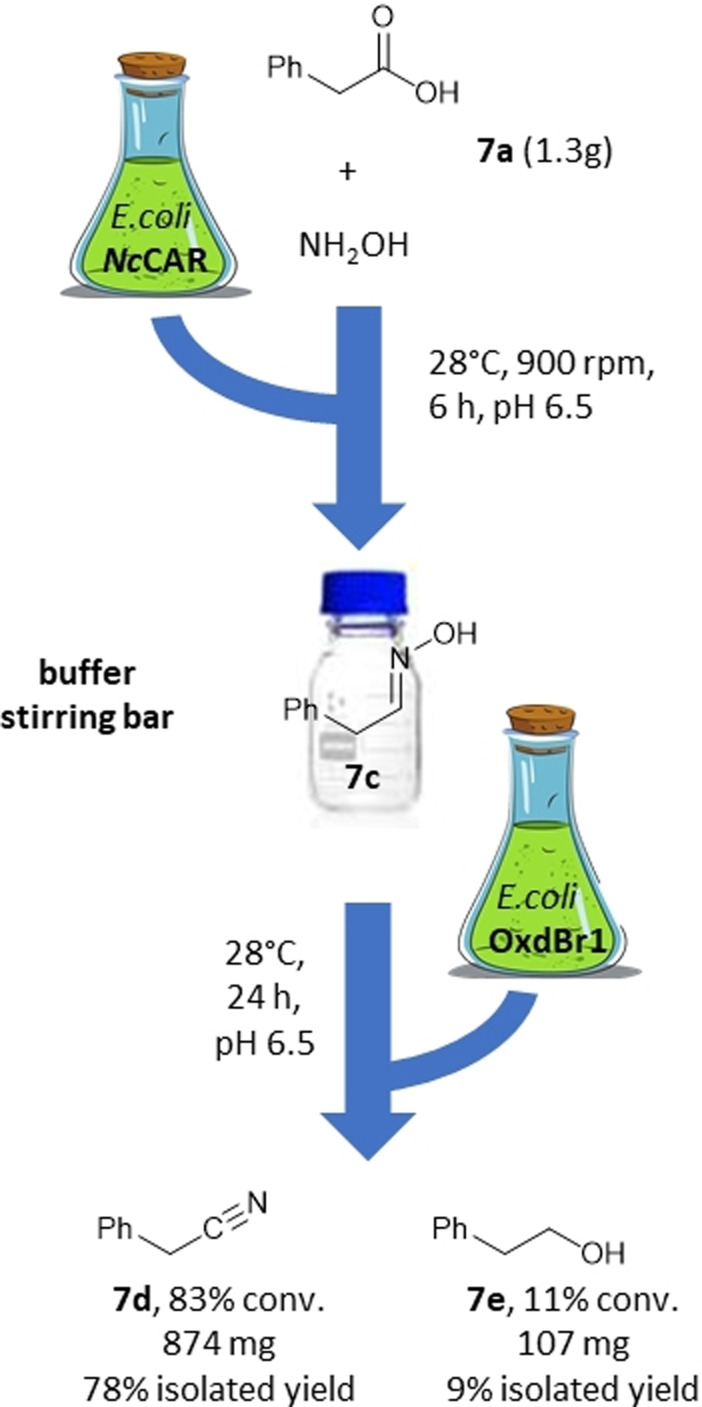
Preparative chemoenzymatic two‐step one‐pot cascade from acid to nitrile.

To show the synthetic utility, we aimed for gram scale reactions with aliphatic **3 a** and aryl‐aliphatic **7 a** as the starting materials. Instead of using an additional *in situ* product removal (ISPR) solvent, the use of nitrile products themselves as second phase would be a highly appealing strategy. For Oxd reactions, this has been successful.^[10]^ To be able to quantify product formation, we used **3 d** as the ISPR solvent for the cascade reaction from **7 a** to **7 d** and **7 d** as the ISPR solvent for **3 d** synthesis. Product titers were, however, lower with nitriles as ISPR solvents (Table 2). We ascribed this effect to inhibition of the CAR by nitriles, because the majority of material was non‐reacted carboxylic acid, which points at a limitation in carboxylate reduction. *Nc*CAR is inhibited by **7 d** *in vitro*.^[12]^ In addition, ATP supply might be limiting because of effects of nitriles on cell viability. Hence, the choice of ISPR solvent for chemoenzymatic synthesis of **3 d** was driven by down‐stream processing considerations. For the two‐step one‐pot reactions we opted for *n*‐hexadecane, which has a sufficiently different boiling point than **3 d**. **3 a** was incubated with *Nc*CAR expressing cells in a magnetically stirred two‐phase system of buffer and *n*‐hexadecane in the presence of hydroxylamine for 6 h. Subsequently, Oxd expressing cells were added and the reaction continued for 24 h. **3 a** was fully consumed, giving 89% of **3 d** and 11% of **3 e**. The isolation of **3 d** appeared to be inefficient from the large volume of *n*‐hexadecane. In the same setup, however, without additional ISPR solvent, **7 a** was converted to **7 d** (Scheme 2). In this case, <2% of residual **7 a** and **7 c**, respectively, were detected at 83% of **7 d**, which was isolated and purified by column chromatography in 78% yield. The respective alcohol **7 e** was isolated as a by‐product in 9% yield.

**Table 2:** One‐pot one‐step cascades with *Nc*CAR and OxdBr1 with nitriles as ISPR solvent in comparison to standard conditions. Substrate (10 mM) in the presence of 1.5 equivalents of hydroxylamine.

Substrate	ISPR solvent	Residual **a** [%]	**∑ b**, **c**, **e** [%]^[a]^	**d** [%]
**3 a**	*n‐*heptane	11	19	69
**3 a**	**7 a**	69	0	31
**7 a**	‐	0	34	66
**7 a**	**3 a**	81	1	18

^[a]^ **e** is a result of competing over‐reduction of the aldehyde through cellular background enzymes.

## Conclusion

In conclusion, a chemoenzymatic route from carboxylic acid to nitrile was developed. The three‐step cascade includes whole‐cell mediated reduction of the carboxylic acid to the respective aldehyde, which is trapped in form of an aldoxime in a chemical step. Finally, the intermediate aldoxime is enzymatically dehydrated to the respective nitrile by Oxd. In order to choose the most promising enzyme combinations for particular substrates, separate screening experiments were performed. A panel of CARs was screened for chemoenzymatic aldoxime synthesis and a panel of Oxds was screened for nitrile formation from aldoxime precursors. The best candidates were combined in one‐pot two‐step reactions. While benzoic acid was converted to nitrile surprisingly well,^[14]^ substituted aromatic acids were reduced by the CAR but not dehydrated efficiently. For aliphatic and aryl‐aliphatic substrates, high yielding CAR/Oxd combinations were identified.

In view of synthetic application of this system, gram scale reactions were carried out. In case of hexanenitrile, experience showed that *in situ* product removal through liquid extraction was beneficial both for conversion and recovery rates.^[12]^ However, nitrile as an ISPR co‐solvent reduced conversions and hexadecane as co‐solvent compromised product isolation. Further optimizations in this direction are necessary, especially for highly volatile short chain aliphatic products. Due to its lower volatility, no extra ISPR solvent was required for phenylacetonitrile synthesis, and the product was isolated in 78% yield.

## Experimental Section

Compounds, if not otherwise stated, were purchased from Sigma Aldrich (Vienna, Austria) with the highest available purities and used without further treatment, except noted otherwise. *E. coli* K‐12 MG1655 RARE (DE3) was kindly provided by K.L.J. Prather.^[25]^ Expression plasmids for producing OxdRG,^[26]^ OxdRE^[27]^ and OxdFG^[28]^ were kindly provided by H. Gröger. Oximes **3 c**, **6 c** and **7 c** were prepared as described previously (SI of Ref^[12]^).

**Strains and Cultivations**: *E. coli* BL21 Star^TM^ (DE3) strains expressing Oxds *via* pET28a(+) were cultivated as described previously.^[23]^ CARs were produced in *E. coli* K‐12 MG1655 RARE (DE3) as described in Horvat *et al*. using the pETDuet vector to co‐express the *E. coli* phosphopantetheinyl transferase (*Ec*PPTase).^[19]^ The empty vector control used for the dehydration reactions was pET28a(+). pETDuet‐ 1:*Ec*PPTase was used as a negative control for the cascade reactions.

**Oxime synthesis from acids using CARs in whole cells**: Reductions were performed with 15 OD_600_ units [approx, 15 mg of cell wet weight (CWW) mL^−1^] in 400 μL of 300 mM HEPES buffer, pH 7.5, containing glucose (100 mM), MgSO_4_ (71 mM), the desired amount of hydroxylamine and 20 mM **a**. Reactions with **1 a**–**3 a** were performed in 15 mL Pyrex tubes in the presence of 400 μL of *n*‐heptane with 0.01% internal standard (IS, *n*‐tetradecane) on a cell culture rotator. Reactions with **4 a**–**8 a** were performed in 96 deep‐well plates (DWPs) under shaking at 320 rpm. Compound **4 a** was dissolved in DMSO and added to the aqueous phase in a final concentration of 5% v/v DMSO. Reactions were performed at 28 °C for 4.5 h. Reactions with **1 a**–**3 a** were terminated by the addition of HCl (3 M, 50 μL). After removing *n*‐heptane by centrifugation, a second extraction with *n*‐heptane (400 μL) was performed, followed by centrifugation. The combined organic layer was dried over Na_2_SO_4_ and analyzed with GC‐ FID. Reactions with **4 a**, **7 a** and **8 a** were terminated by the addition of HCl (3 M, 50 μL), and extracted with ethyl acetate (2×400 μL). The combined organic layer was dried over Na_2_SO_4_ and analyzed with GC‐FID. Reactions with **5 a** were terminated by the addition of 800 μL MeOH. Reactions with **6 a** were terminated by the addition of 800 μL EtOH. After mixing well, DWPs were centrifuged for 1 h at 4,000 rpm. The clear supernatants were analyzed by HPLC‐UV.

**Nitrile synthesis from oximes using Oxds in whole cells**: Biodehydrations were performed with 10 OD_600_ units in 1 mL of 300 mM HEPES buffer, pH 7.5. Expression levels of wt and mutant CARs (typeI and typeIII), and *Pc*CAR4 were similar (high) while *Tv*CAR and *Pc*CAR2 showed lower levels and *Tv*CAR2 the lowest expression level. Substrates were dissolved in DMSO and added to the aqueous phase. 1 mL of *n*‐heptane with 0.01% internal standard (IS, *n*‐tetradecane) or *n‐*hexadecane with 0.01% IS (*n‐*heptane) was used as second layer for aliphatic compounds. Biotransformations were performed in 15 mL Pyrex tubes on a cell culture rotator at 28 °C for 1 h. Aromatic substrates with low conversion rates were incubated overnight. Reactions were terminated and analyzed as described for oxime synthesis above.

**Chemoenzymatic cascades with CARs and Oxds in the presence of hydroxylamine**: The aqueous phase (1 mL) consisted of glucose (100 mM), MgSO_4_ (71 mM) and NH_2_OH (15 mM) in HEPES buffer (300 mM, pH 7.5) and 20 OD_600_ units of *E. coli* MG1655 RARE (DE3) cells expressing CAR. For two‐phase cascade reactions, the organic layer containing carboxylate **1 a**–**3 a** (10 mM) dissolved in *n‐*heptane (1 mL, 0.01% IS) was added to 1 mL of aqueous reaction mixture in 15 mL Pyrex tubes. Single phase reactions were carried out in DWPs with 500 μL of the above‐described reaction mixture, with 10 mM of **4 a**–**8 a**. The oxime formation step was carried out for 4 h in a culture tube rotator (**1 a**–**3 a**) or titramax (320 rpm, **4 a**–**8 a**) at 28 °C. Dehydration of **c** to **d** was initiated by addition of 10 OD_600_ units of Oxd in 20 μL of reaction buffer without NH_2_OH. The reactions were incubated for additional 20 h. Reactions were terminated and analyzed as described for oxime synthesis above.

**Gram scale Chemoenzymatic cascade**: *E. coli* MG1655 RARE (DE3) cells expressing CAR were suspended in MES buffer (300 mM, pH 6.5; 5×200 mL in stirred bottles, OD_600_=20) with glucose (100 mM), MgSO_4_ (71 mM) and NH_2_OH (15 mM). The reaction was started with **7 a** (in total 1.3 g dissolved in KOH). After 6 h at 28 °C and stirring at 900 rpm, *E. coli* BL21 star (DE3) expressing OxdBr1 was added (OD_600_=10). The reaction was allowed to stir for 24 h at 28 °C. GC analysis revealed 1.5% residual **7 a**, 1.3% **7 c**, 11.3% **7 e** and 82.9% desired **7 d**. The reaction was terminated by the addition of HCl. Compounds were extracted twice with ethyl acetate and purified by column chromatography. **7 d** was obtained as light‐yellow liquid (874 mg, 78% yield). **7 e** was obtained as colorless liquid (107 mg, 9% yield).

Analytical methods are described in the Supporting Information.

## Supporting information

As a service to our authors and readers, this journal provides supporting information supplied by the authors. Such materials are peer reviewed and may be re‐organized for online delivery, but are not copy‐edited or typeset. Technical support issues arising from supporting information (other than missing files) should be addressed to the authors.

Supporting Information
