# Supplementary material for: Organic Acid to Nitrile: A Chemoenzymatic Three‐Step Route
Source: Adv Synth Catal. 2022 Dec 28;365(1):37–42. doi: 10.1002/adsc.202201053 (PMC10107818; doi:10.1002/adsc.202201053)
Supplement: Supplementary file 1 — Supporting Information [file ADSC-365-37-s001.pdf]

Advanced Synthesis & Catalysis

Supporting Information

Organic Acid to Nitrile: A Chemoenzymatic Three-Step Route

Margit Winkler,* Melissa Horvat, Astrid Schiefer, Victoria Weilch, Florian Rudroff, Miroslav Pátek, and Ludmila Martínková*© 2022 The Authors. Advanced Synthesis & Catalysis published by Wiley-VCH GmbH. This is an open access article under the terms of the Creative Commons Attribution License, which permits use, distribution and reproduction in any medium, provided the original work is properly cited.

Supplementary information

Organic Acid to Nitrile: a Chemoenzymatic Three-Step Route

Margit Winkler, Melissa Horvat, Astrid Schiefer, Victoria Weilch, Florian Rudroff, Miroslav Pátek, Ludmila Martínková,

Contents

1. Additional data and figures 2

1.1 CAR mediated enzymatic transformations from acid to aldehyde with in situ chemical trapping to oxime 2

2. Materials and Methods 4

2.1 List of enzymes 4

2.2 Gram scale Chemoenzymatic cascade to hexanenitrile 3d 5

2.3 GC-FID analysis 6

2.4 HPLC-UV analysis 6

2.5 Butyraldehyde oxime (**1c**) synthesis and purification 6

2.6 Pentanal oxime (**2c**) synthesis and purification 7

2.7 Hexanal oxime (**3c**) synthesis and purification 7

2.8 3-phenylpropanal oxime (**8c**) synthesis and purification 7

3. NMR spectra 8

4. References 15

1. Additional data and figures

1.1 CAR mediated enzymatic transformations from acid to aldehyde with in situ chemical trapping to oxime

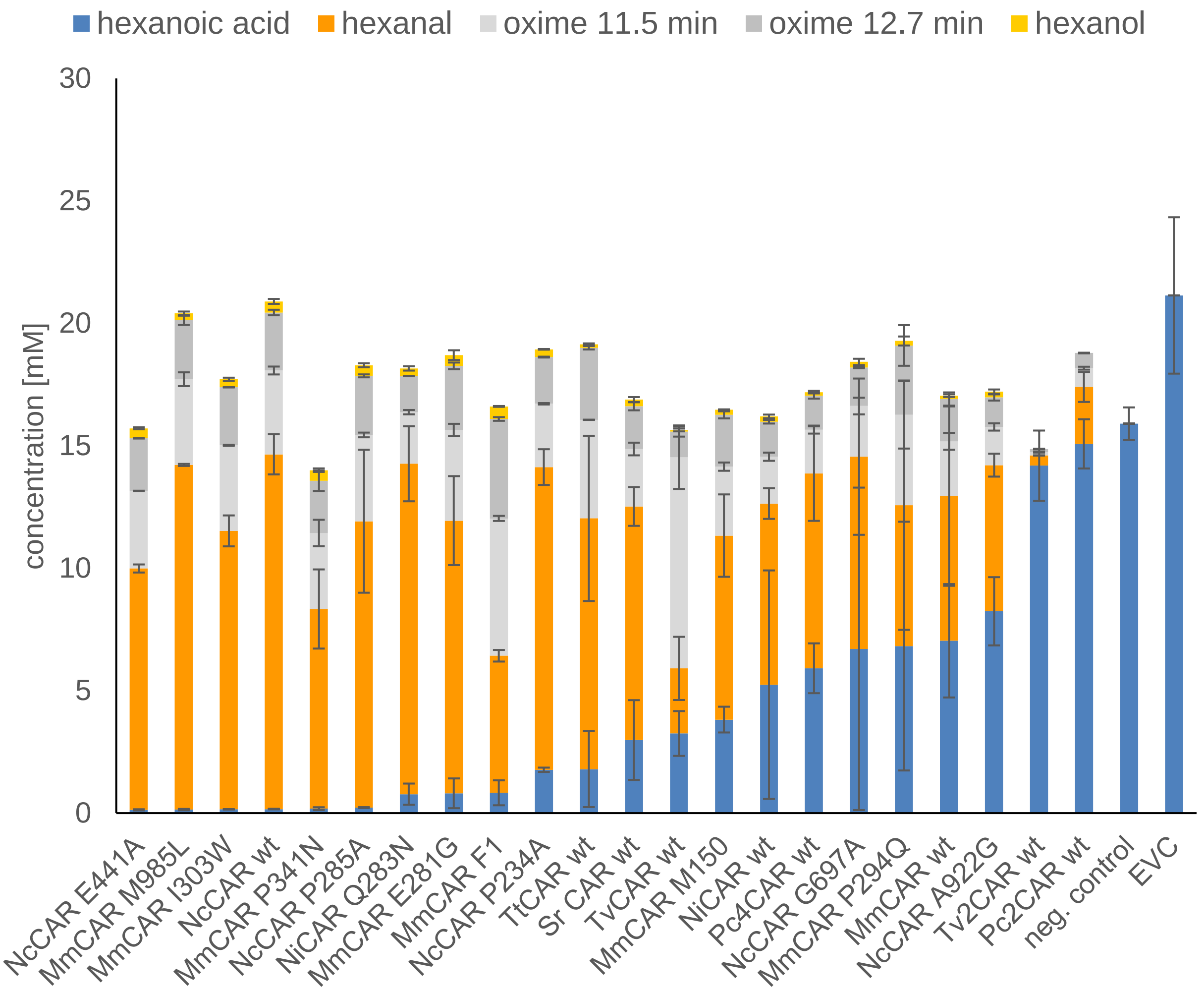

Figure S1. Chemoenzymatic oxime formation from hexanoic acid to *E*/*Z* hexanal oxime 3c. Whole cell biotransformations with 20 mM **3a** as the substrate in the presence of 30 mM NH_2OH and *n*-heptane for *in situ* product removal. Reaction time: 4.5 h at 28°C.Error bars are shown for technical duplicates. Samples were analyzed by GC/FID. EVC = empty vector control. wt = wild type.

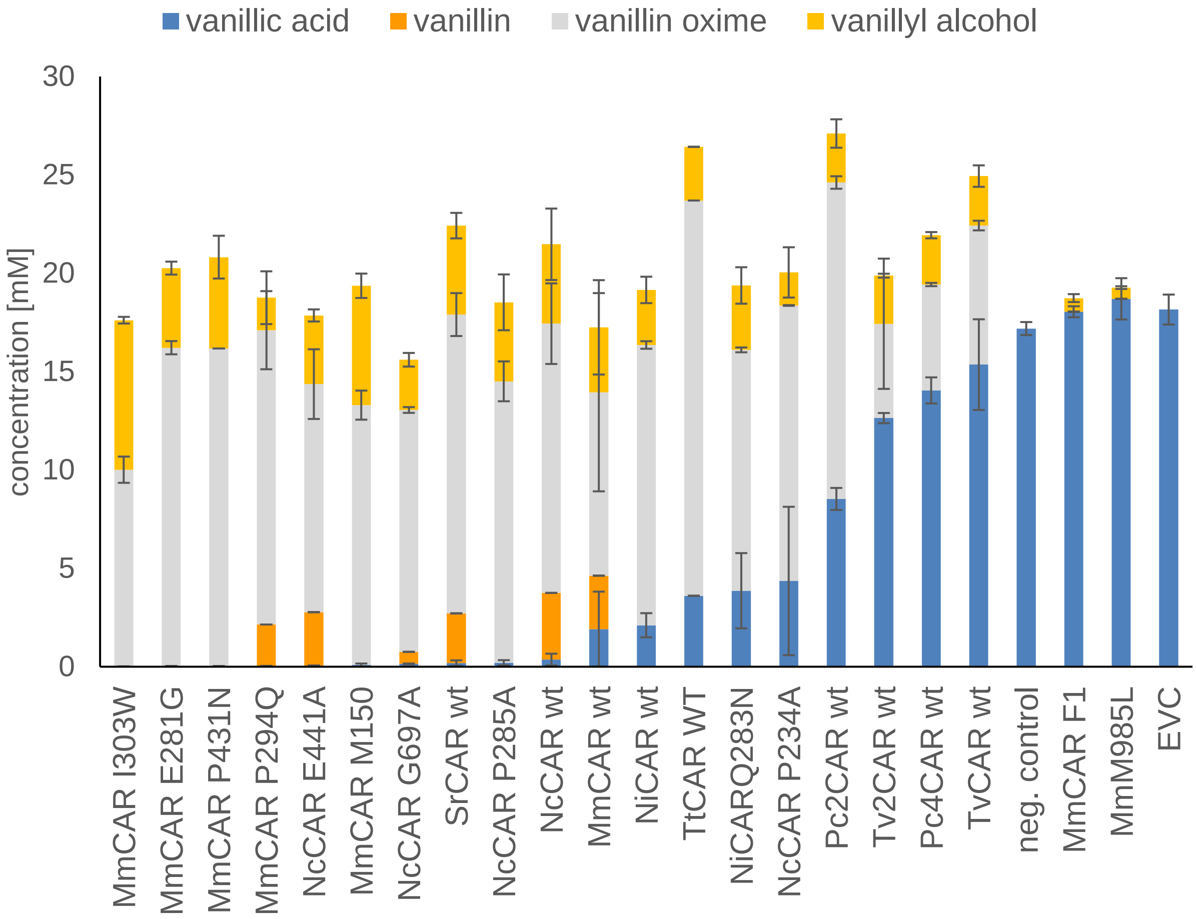

Figure S2. Chemoenzymatic oxime formation from vanillic acid (5a) to *E*-vanillin oxime (5c). Whole cell biotransformations with 20 mM **5a** as the substrate in the presence of 30 mM NH_2OH. Reaction time: 4.5 h at 28°C.Error bars are shown for technical duplicates. Samples were analyzed by HPLC/UV. EVC = empty vector control. wt = wild type.

2. Materials and Methods

2.1 List of enzymes

Enzyme Abbreviation	Organism	Variant	Accession Number NCBI	Reference
*Tt*CAR	*Thermothelomyces thermophila*		XP_003665695.1	[1]
*Tv*CAR	*Trametes versicolor*		XP 008043822.1	[2]
*Tv2*CAR	*Trametes versicolor*		XP_008044321.1	[3]
*Sr*CAR	*Segniliparus rotundus*		WP 013138593.1	[4]
*Ni*CAR	*Nocardia iowensis*		Q6RKB1.1	[5]
*Ni*CAR Q283N	*Nocardia iowensis*	Q283N	Q6RKB1.1	[6]
*Pc2*CAR	*Pycnoporus cinnabarinus*		A0A060SHA6	[7]
*Pc4*CAR	*Pycnoporus cinnabarinus*		A0A060SU16	[7]
*Mm*CAR	*Mycobacterium marinum*		WP_012393886	[8]
*Mm*CAR P294Q	*Mycobacterium marinum*	P294Q	WP_012393886	[9]
*Mm*CAR E281G	*Mycobacterium marinum*	E281G	WP_012393886	[9]
*Mm*CAR P341N	*Mycobacterium marinum*	P341N	WP_012393886	[9]
*Mm*CAR F1	*Mycobacterium marinum*	C983P, D984H, M985L	WP_012393886	[10]
*Mm*CAR M150	*Mycobacterium marinum*	D241E, H454R, L567M	WP_012393886	[10]
*Mm*CAR M985L	*Mycobacterium marinum*	M985L	WP_012393886	[10]
*Mm*CAR I303W	*Mycobacterium marinum*	I303W	WP_012393886	[10]
*Nc*CAR	*Neurospora crassa*		XP_955820.1	[11,12]

*Nc*CAR P234A	*Neurospora crassa*	P234A (M6)	XP_955820.1	[13]
*Nc*CAR P285A	*Neurospora crassa*	P285A (M18)	XP_955820.1	[13]
*Nc*CAR E441A	*Neurospora crassa*	E441A (M22)	XP_955820.1	[13]
*Nc*CAR G697A	*Neurospora crassa*	G697A (M29)	XP_955820.1	[13]
*Nc*CAR A922G	*Neurospora crassa*	A922G (M33)	XP_955820.1	[13]
OxDFv	*Fusarium vannetenii* strain 77-13-4		XP_003042958.1	[14]
OxDBr1	*Bradyrhizobium sp.* LTSPM299		WP_044589203.1	[15]
OxDCp	*Corynebacterium pacaense* Marseille-P2417		WP_080796375.1	This work

2.2 Gram scale Chemoenzymatic cascade to hexanenitrile 3d

E. coli MG1655 RARE (DE3) cells expressing CAR were suspended in MES buffer (300 mM, pH 6.5; 5 x 200 mL in stirred bottles, OD_{600} = 20) with glucose (100 mM), $MgSO_4$ (71 mM) and NH_2OH (15 mM). The reaction was started with **3a** (220 mg in 200 mL *n*-hexadecane per reaction bottle). After 6 h at 28°C and stirring at 900 rpm, *E. coli* BL21 star (DE3) expressing OxdBr1 was added (OD_{600} = 10).[15] The reaction was allowed to stir for 24 h at 28°C. GC analysis revealed no residual **3a**, or **3c**, 11.1% **3e** and 88.9% desired **3d**. The reaction was terminated by the addition of HCl. Compounds were extracted with *n*-hexadecane and purified by column chromatography (silica gel 60 with NEt_3). Unfortunately, **3d** co-eluted with the hexadecane. However, **3e** was retained and could be eluted using hexane. **3e** was obtained as colorless liquid (87 mg, 9% yield). The residual hexadecane was distilled using a Vigreux column and **3d** was obtained as colorless liquid (53 mg, 5.8% yield) at 103°C (150 mbar).

3d: ^{1}H NMR (400 MHz, Chloroform-*d*) δ 0.92 (t, *J* = 7.2 Hz, 3H, CH_3), 1.28 – 1.49 (m, 4H, CH_2), 1.60 – 1.72 (m, 2H, CH_2), 2.33 (t, *J* = 7.2 Hz, 2H, CH_2CN).

^{13}C NMR (101 MHz, Chloroform-*d*) δ 13.9 (C6), 17.2 (C2), 22.0 (C5), 25.2 (C3), 30.9 (C4), 120.0 (C1).

3e: ^{1}H NMR (400 MHz, Chloroform-*d*) δ 0.80 – 1.02 (m, 3H, CH_3), 1.14 – 1.46 (m, 6H, CH_2), 1.56 (tt, *J* = 8.0, 6.1 Hz, 2H, CH_2), 1.71 (s, 1H, OH), 3.63 (td, *J* = 6.6, 4.7 Hz, 2H, CH_2OH).

^{13}C NMR (101 MHz, Chloroform-*d*) δ 14.2 (C6), 22.8 (C3/C4/C5), 25.6 (C3/C4/C5)), 31.8 (C3/C4/C5), 32.9 (C2), 63.2 (C1).

2.3 GC-FID analysis

For GC-FID measurements, a ZP-5 column (crosslinked 5% Ph-Me Siloxane; 30 m, 0.32 mm diameter, 0.25 µm film thickness) on a Shimadzu GC 2030 equipped with an FID was used. Sample aliquots of 1 µL were injected in split mode (split ratio 10:1) at 240°C injector temperature and 320°C detector temperature with N_2 as carrier gas. The temperature gradient for **3a**, **3b**, ***E*/*Z*-3c**, **3d** and **3e** as well as **7a**, **7b**, ***E*/*Z*-7c**, **7d** and **7e** was reported previously[16] and **4a**, **4b**, ***E*/*Z*-4c**, **4d** and **4e** were also analyzed with this method. The temperature gradient for **1a**, **1b**, ***E*/*Z*-1c**, **1d** and **1e** started with a hold at 50°C for 5 min, followed by temperature gradients to 80°C at 5°C min^{-1} and then to 300°C at 40°C min^{-1} and a hold at 300°C for 2 min. The total run time was 18.5 min. The temperature gradient for **2a**, **2b**, ***E*/*Z*-2c**, **2d** and **2e** started with a hold at 60°C for 6 min, followed by temperature gradients to 100°C at 10°C min^{-1} and then to 300°C at 40°C min^{-1} and a hold at 300°C for 2 min. The total run time was 17.0 min. The temperature program for quantification of **8a**, **8b**, ***E*/*Z*-8c**, **8d** and **8e** started with a hold at 70°C for 3 min, followed by a temperature gradient to 250°C at 40°C min^{-1} and a hold for 5 min, continued by a temperature ramp to 300°C at 40°C min^{-1} and a hold for 3 min. The total runtime was 16.75 min. GC-FID results were evaluated with the GC-FID Data Analysis software LabSolution (Shimadzu). Quantification of all compounds was established through linear intrapolation from calibration curves with authentic standard.

2.4 HPLC-UV analysis

HPLC-UV analysis for compounds **5**, **6** and derivatives were performed according to the methods previously reported.[1,17]

*2.5 Butyraldehyde oxime (**1c**) synthesis and purification*

Butyraldehyde oxime **1c** was synthesized according to Hinzmann *et al*.[20] **1b** was distilled prior to use. A RBF (250 mL) was charged with sodium carbonate (12.26 g, 115.7 mmol, 0.75 equiv.) which was dissolved in water (285 mL) and ethanol (15 mL). Subsequently, hydroxylamine hydrochloride (16.19 g, 233 mmol, 1.5 equiv.) and **1b** (11.1 g, 154 mmol, 1 equiv.) were added. Vacuum was applied to the flask for a short time and then the flask was purged with argon. The reaction solution was stirred at rt under Ar atmosphere overnight (19 h). NMR analysis of the reaction mixture confirmed full conversion. The ethanol was removed by rotary evaporation. Subsequently the aqueous layer was extracted with diethyl ether (3 x 150 mL). The combined organic layers were washed with brine (150 mL) and then dried over Na_2SO_4. The solvent was removed by rotary evaporation to yield 6.57 g (50 %) **1c** as colorless liquid with an *E*/*Z*-ratio of 55/45.

^{1}H NMR (400 MHz, Chloroform-*d*) δ 0.96 (q, *J* = 7.3 Hz, 6H, H4), 1.53 (hd, *J* = 7.3, 1.0 Hz, 4H, H3), 2.18 (td, *J* = 7.4, 6.1 Hz, 2H, E-H2), 2.37 (td, *J* = 7.5, 5.5 Hz, 2H, Z-H2), 6.73 (t, *J* = 5.5 Hz, 1H, Z-H1), 7.42 (t, *J* = 6.1 Hz, 1H, E-H1), 8.53 (s, 2H, -OH).

^{13}C NMR (101 MHz, Chloroform-*d*) δ 13.7 (E-C4), 14.0 (Z-C4), 19.6 (Z-C3), 20.0 (E-C3), 27.0 (Z-C2), 31.5 (E-C2), 152.3 (E-C1), 152.9 (Z-C1).

*2.6 Pentanal oxime (**2c**) synthesis and purification*

Pentanal oxime **2c** was synthesized according to Hinzmann *et al.*[20] **2b** was distilled prior to use. A RBF (250 mL) was charged with sodium carbonate (2.84 g, 26.7 mmol, 0.75 equiv.) which was dissolved in water (90 mL) and ethanol (4.75 mL). Subsequently, hydroxylamine hydrochloride (3.81 g, 54.8 mmol, 1.50 equiv.) and **2b** (3.16 g, 35.6 mmol, 1.00 equiv.) were added. The reaction solution was stirred for 18 h at rt under argon atmosphere during which an oily layer was formed at the surface. As TLC analysis (LP:EA = 25:1, stained with $KMnO_4$) showed incomplete conversion, a second portion of ethanol (5 mL) was added. After additional 4 h, TLC analysis confirmed full conversion. The aqueous solution was extracted with diethyl ether (3 x 100 mL). The combined organic layers were washed with brine (100 mL) and then dried over Na_2SO_4. The solvent was removed by rotary evaporation and a clear, oily residue was obtained. The crude product was purified by flash chromatography (DCM + 1 % MeOH, 80 g silica gel 60) to yield 1.05 g (29 %) **2c** as colorless crystals with an *E*/*Z*-ratio of 54/46.

^{1}H NMR (400 MHz, Chloroform-*d*) δ 0.92 (td, *J* = 7.2, 5.1 Hz, 6H, H5), 1.14 – 1.78 (m, 8H, H3, H4), 2.20 (td, *J* = 7.4, 6.2 Hz, 2H, E-H2), 2.39 (td, *J* = 7.5, 5.5 Hz, 2H, Z-H2), 6.72 (t, *J* = 5.4 Hz, 1H, Z-H1), 7.42 (t, *J* = 6.1 Hz, 1H, E-H1), 8.19 (s, 2H, OH).

^{13}C NMR (101 MHz, Chloroform-*d*) δ 13.9 (C5), 22.3 (E-C4), 22.6 (Z-C4), 24.8 (Z-C2), 28.3 (Z-C3), 28.7 (E-C3), 29.3 (E-C2), 152.4 (E-C1), 153.0 (Z-C1).

*2.7 Hexanal oxime (**3c**) synthesis and purification*

Hexanal oxime **3c** was synthesized according to Hinzmann *et al.*[19] **3b** was distilled prior to use. A RBF (250 mL) was charged with sodium carbonate (7.21 g, 68.0 mmol, 0.75 equiv.) was dissolved in water (142.5 mL) and ethanol (7.5 mL). Subsequently, hydroxylamine hydrochloride (9.54 g, 137 mmol, 1.50 equiv.) and **3b** (9.02 g, 90.1 mmol, 1.00 equiv.) were added. The reaction was stirred for 19 h at rt under argon atmosphere during which an oily layer was formed at the surface. TLC analysis (LP, stained with $KMnO_4$) confirmed full conversion. The aqueous solution was extracted with diethyl ether (3 x 100 mL). The organic layer was washed with brine (100 mL) and then dried over Na_2SO_4 and concentrated to yield a clear, oily residue. The crude product was purified by flash chromatography (CH_2Cl_2 + 1 % MeOH) and yielded 7.67 g of **3c** (74 %) as colorless crystals with an *E*/*Z*-ratio of 55/45.

^{1}H NMR (400 MHz, Chloroform-d) δ 0.56 – 1.05 (m, 6H, H6), 1.18 – 1.42 (m, 8H, H4, H5), 1.48 (dtd, J = 8.5, 7.1, 3.4 Hz, 4H, H3), 2.19 (dtd, J = 519.6, 7.5, 6.2 Hz, 2H, E-H2), 2.37 (td, J = 7.6, 5.4 Hz, 2H, Z-H2), 6.71 (t, J = 5.5 Hz, 1H, Z-H1), 7.42 (t, J = 6.1 Hz, 1H, E-H1), 9.00 (s, 2H, OH).

^{13}C NMR (101 MHz, Chloroform-d) δ 14.0 (d, C6), 22.5 (C5), 25.1 (Z-C2), 25.8 (Z-C3), 26.3 (E-C3), 29.6 (E-C2), 31.3 (E-C4), 31.6 (Z-C4), 152.4 (E-Z1), 153.0 (Z-C1).

*2.8 3-phenylpropanal oxime (**8c**) synthesis and purification*

3-Phenylpropanal oxime **8c** was synthesized according to Hinzmann *et al.* [19] **8b** was distilled prior to use. A RBF (250 mL) was charged with sodium carbonate (5.34 g, 50.4 mmol, 0.75 equiv.) which was dissolved in water (95 mL) and ethanol (5 mL). Subsequently, hydroxylamine hydrochloride (7.22 g, 104 mmol, 1.50 equiv.) and **8b** (9.01 g, 67.1 mmol, 1.00 equiv.) were added. The reaction was stirred for 20 h at rt under argon atmosphere during which precipitation of the product occurred. TLC analysis (LP:EA = 1:1, stained

with $KMnO_4$) confirmed full conversion. The suspension was poured onto ice-water (about 100 mL) and the precipitate was separated via suction filtration. The obtained solids were washed with water (3 x 20 ml) and were then recrystallized from ligroin (108 mL) to yield 9.47 g (95 %) of **8c** as colorless crystals with an *E*/*Z*-ratio of 52/48.

^{1}H NMR (600 MHz, Chloroform-*d*) δ 2.48 – 2.61 (m, 2H, E-H2), 2.69 – 2.79 (m, 2H, Z-H2), 2.84 (td, *J* = 7.9, 4.9 Hz, 4H, H3), 6.77 (t, *J* = 5.3 Hz, 1H, Z-H1), 7.15 – 7.25 (m, 6H, H5, H7, H9), 7.28 – 7.37 (m, 4H, H6, H8), 7.47 (t, *J* = 5.9 Hz, 1H, E-H1).

^{13}C NMR (151 MHz, Chloroform-*d*) δ 26.5 (Z-C2), 31.3 (E-C2), 32.1 (Z-C3), 32.9 (E-C3), 126.4 (C7), 128.4 (C5, C9), 128.5 (C5, C9), 128.7 (C6, C8), 140.6 (E-C4), 140.8 (Z-C4), 151.5 (E-C1), 151.9 (Z-C1).

2.8 Cultivation of E. coli *BL21 (DE3) expressing aldoxime dehydratase OxdFv or OxdCp*

Aldoxime dehydratases OxdFv (EEU37245) and OxdCp (WP_080796375) were expressed in *E. coli* BL21 (DE3) harbouring vector pET-28a(+) with the corresponding genes (optimized and synthetized by GeneArt, Thermofisher Scientific) ligated between *Nde*I and *Xho*I sites. The cultivation conditions were 2xYT medium with 30 µg kanamycin/mL (250 mL medium per 500-mL non-baffled flask), 30 °C, 150 rpm. When OD_{600} reached 1.0, gene expression was induced with 0.1 mM IPTG, the cultivation temperature was decreased to 16 °C and the cultivation was continued for 24 h. Harvested cells were used immediately or stored at -80 °C.

3. NMR spectra

All NMR spectra were measured at 400 MHz using Bruker Avance Ultrashield 400 spectrometer. The chemical shifts are given in ppm, and the coupling constants are given in Hz. For describing the multiplicities of the peaks, the following abbreviations were used: s = singlet; d = doublet; t = triplet; q = quartet; m = multiplet; bs = broad singlet. 13C NMR experiments were measured with proton decoupling.

Butyraldehyde oxime **1c**

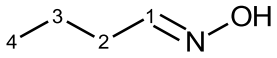

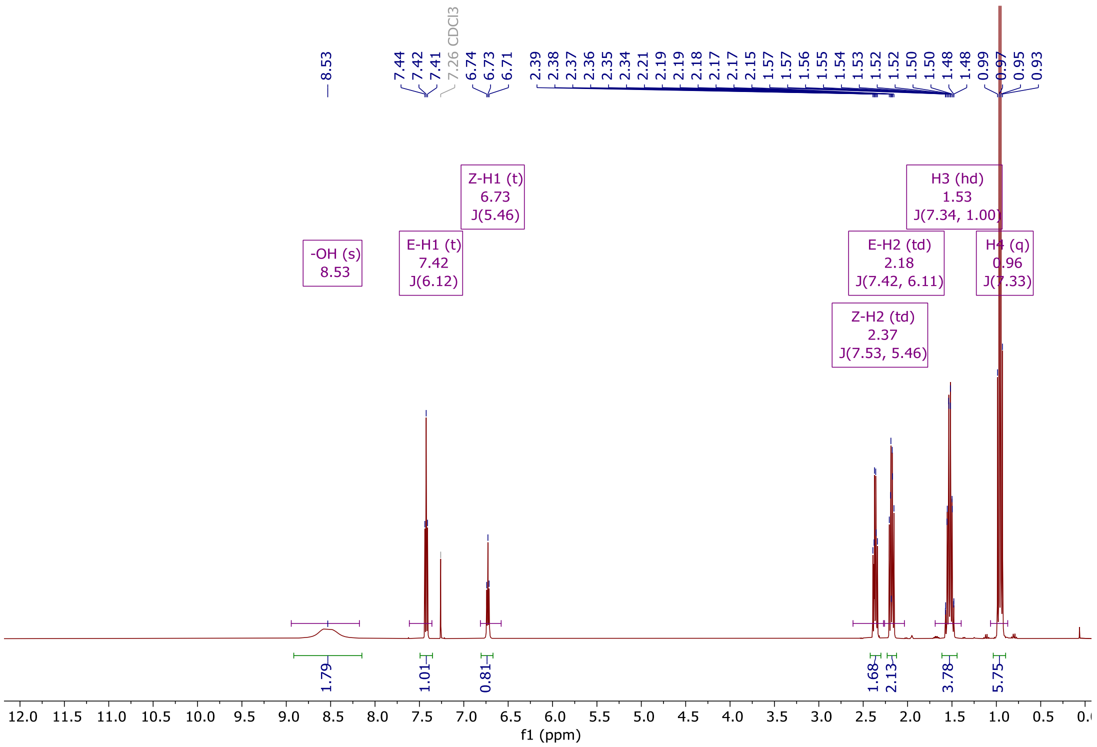

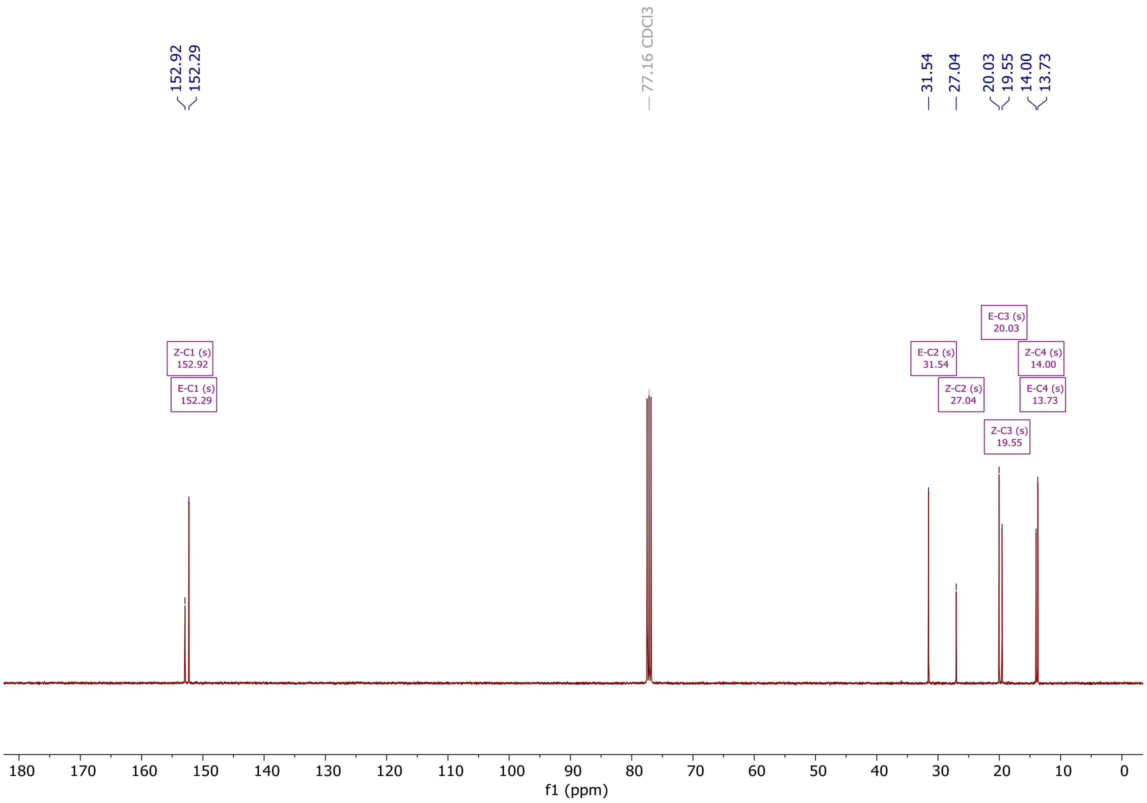

Pentanal oxime **2c**

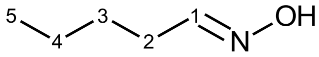

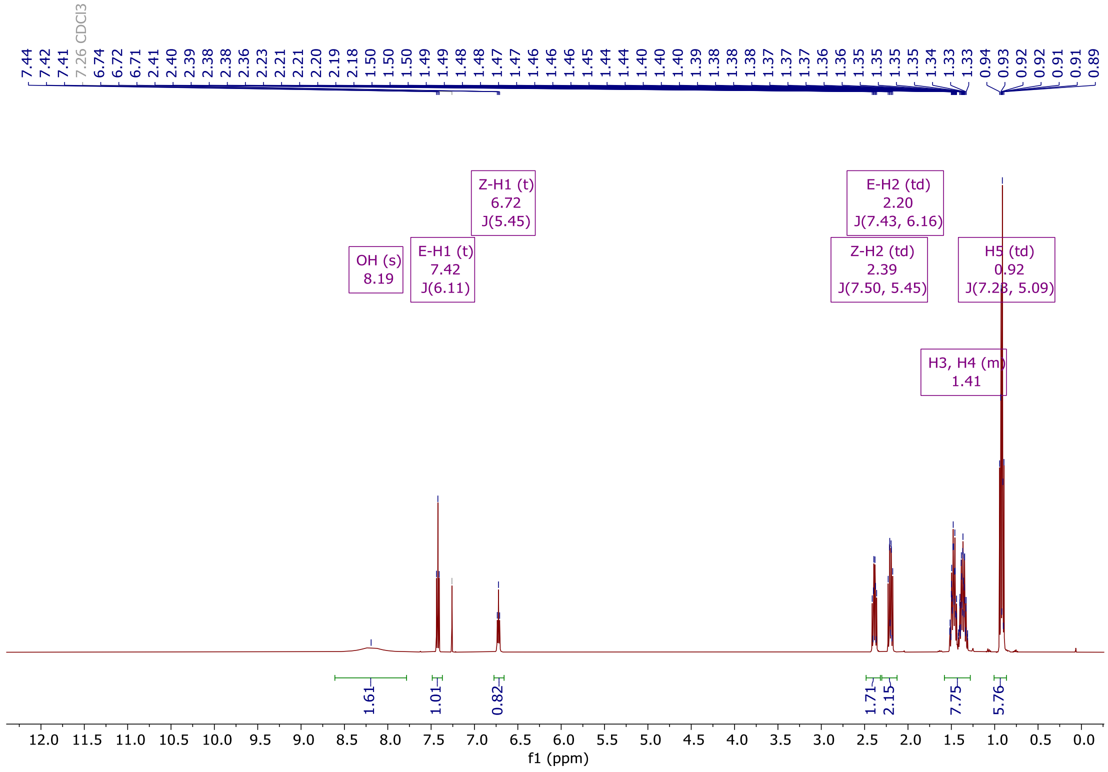

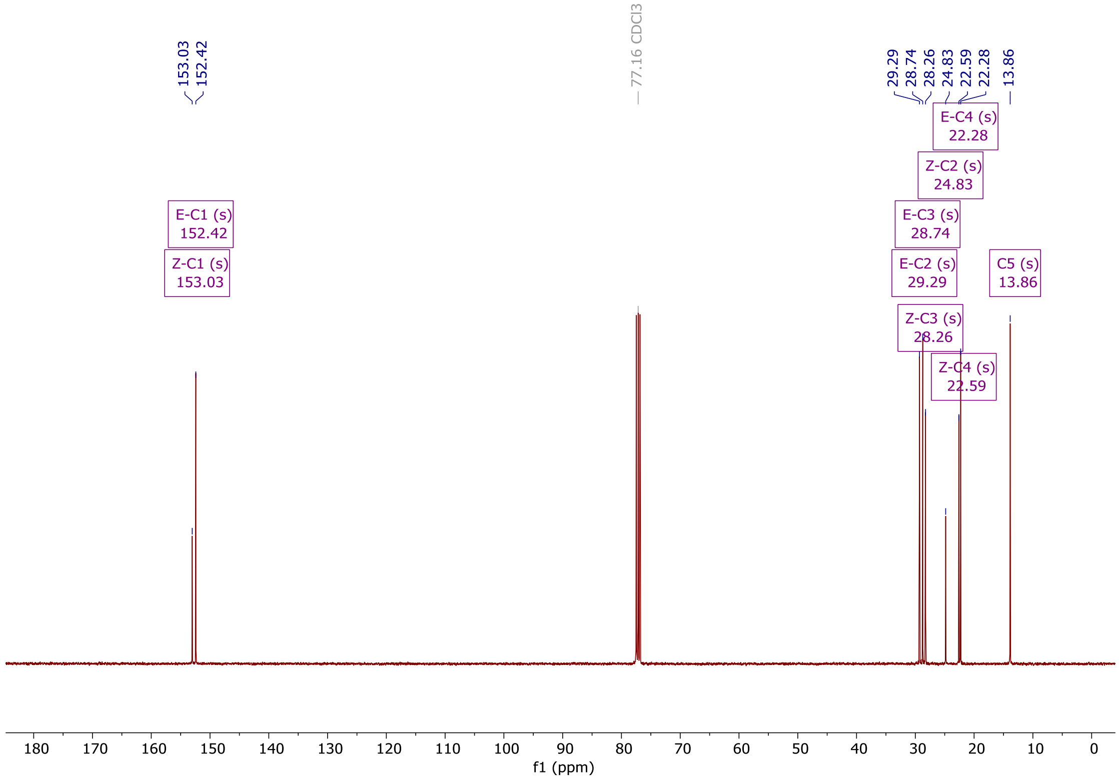

Hexanal oxime **3c**

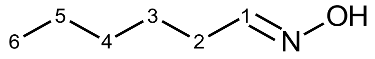

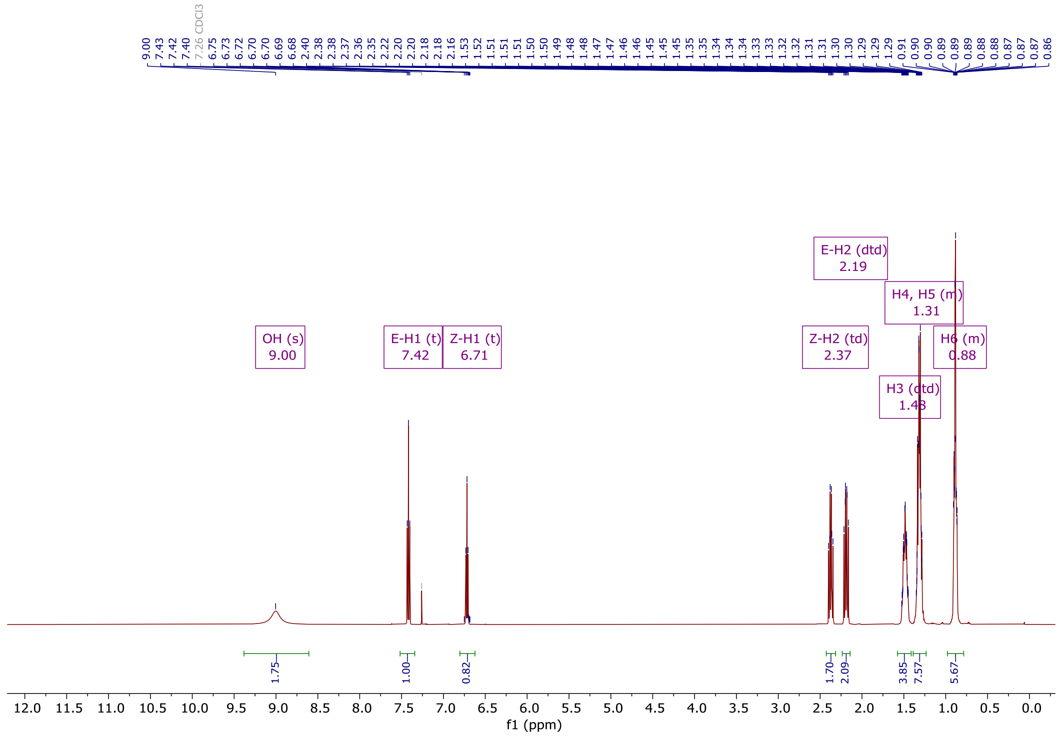

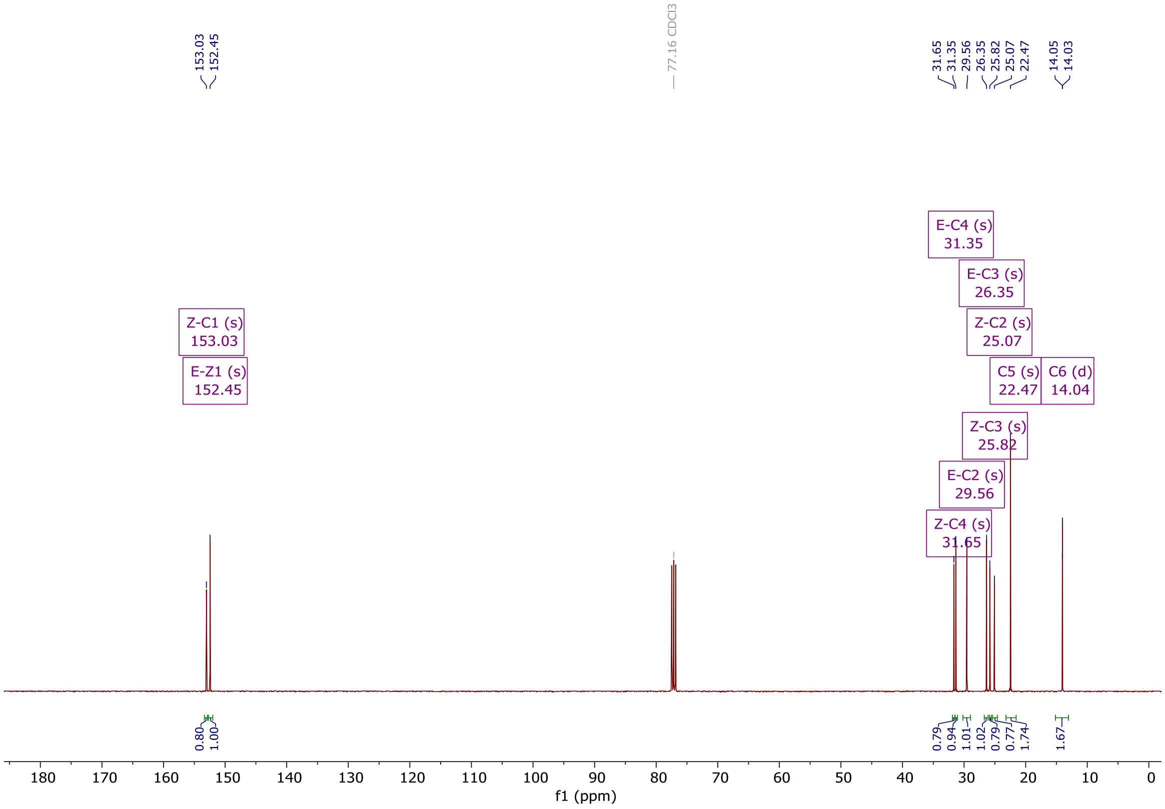

Hexanenitrile **3d**

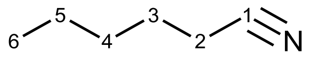

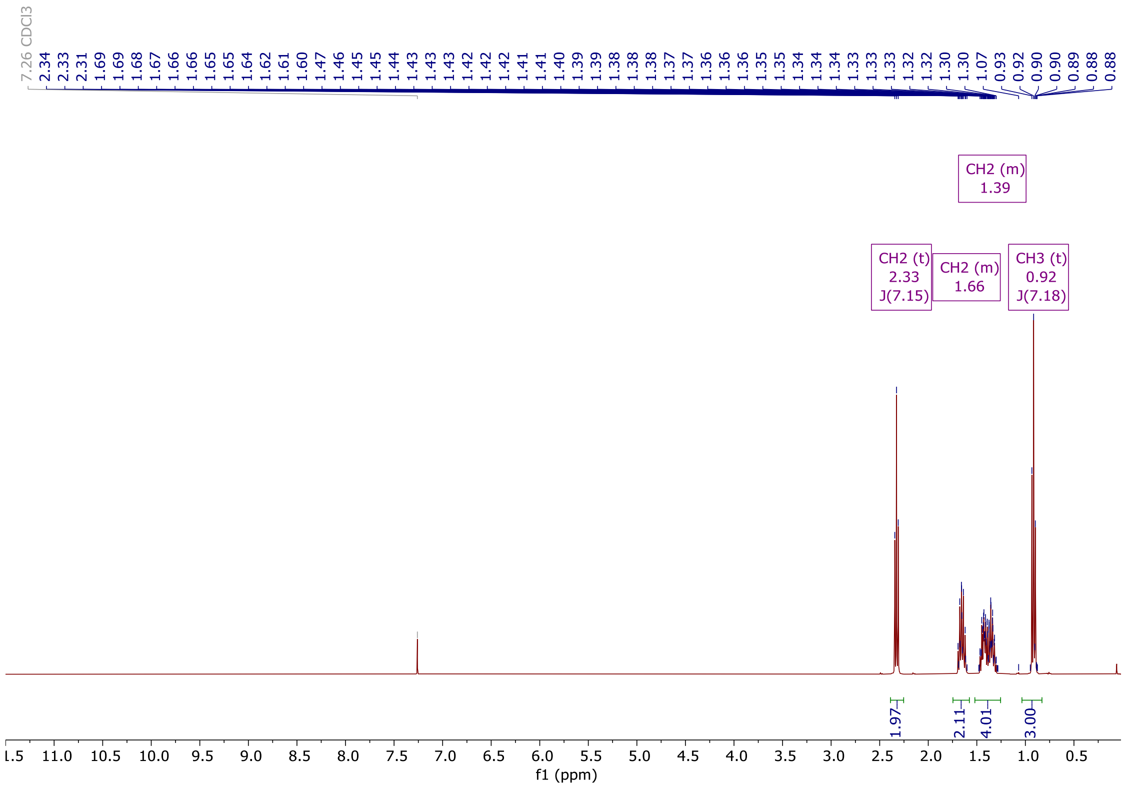

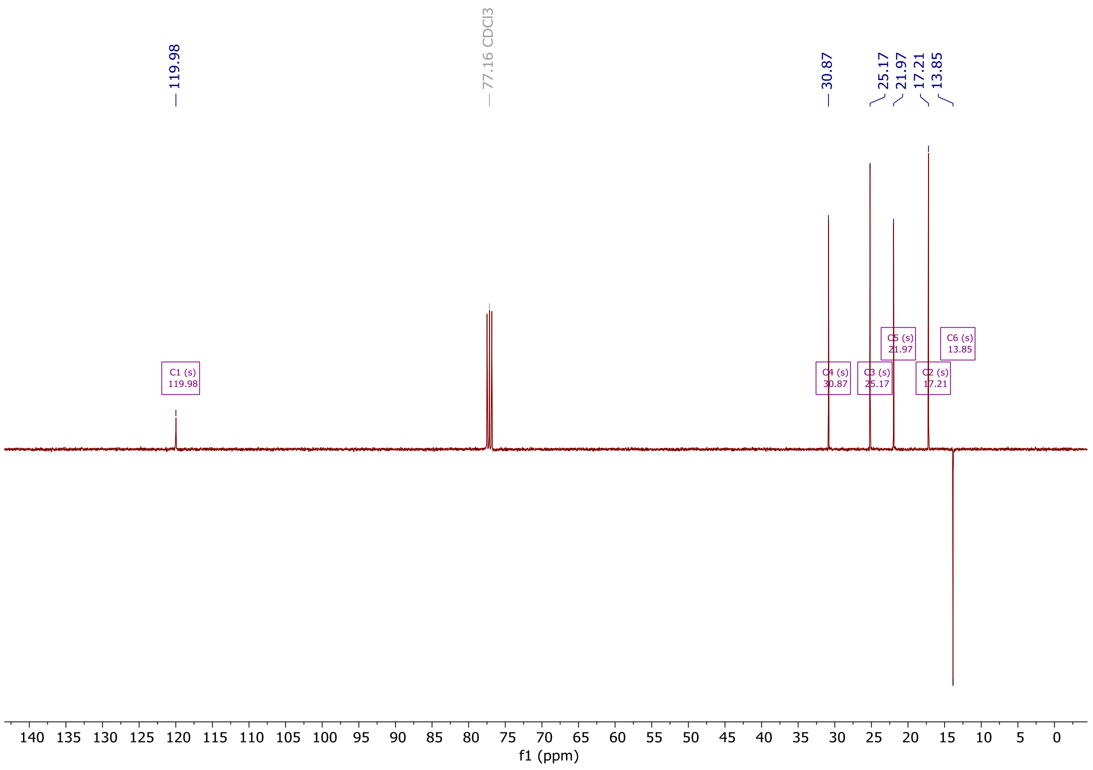

Hexanol **3e**

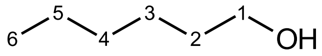
6
5
4
3
2
1
OH

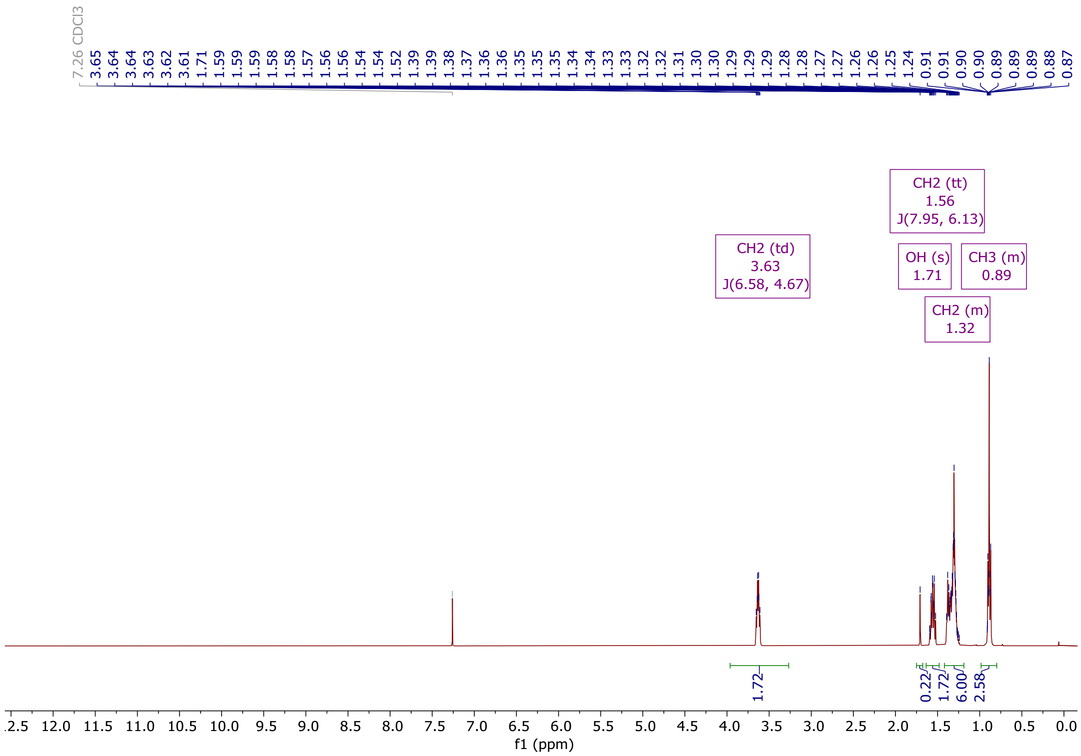
7.26 CDCl3
3.65 3.64 3.64 3.63 3.62 3.61 1.71 1.59 1.59 1.59 1.58 1.58 1.57 1.56 1.56 1.54 1.54 1.52 1.39 1.39 1.38 1.37 1.36 1.36 1.35 1.35 1.35 1.34 1.34 1.33 1.33 1.32 1.32 1.31 1.30 1.30 1.29 1.29 1.28 1.28 1.27 1.27 1.26 1.25 1.24 0.91 0.91 0.90 0.90 0.89 0.89 0.89 0.88 0.87
CH2 (tt) 1.56 J(7.95, 6.13)
CH2 (td) 3.63 J(6.58, 4.67)
OH (s) 1.71
CH3 (m) 0.89
CH2 (m) 1.32
1.72
0.22
1.72
6.00
2.58
f1 (ppm)

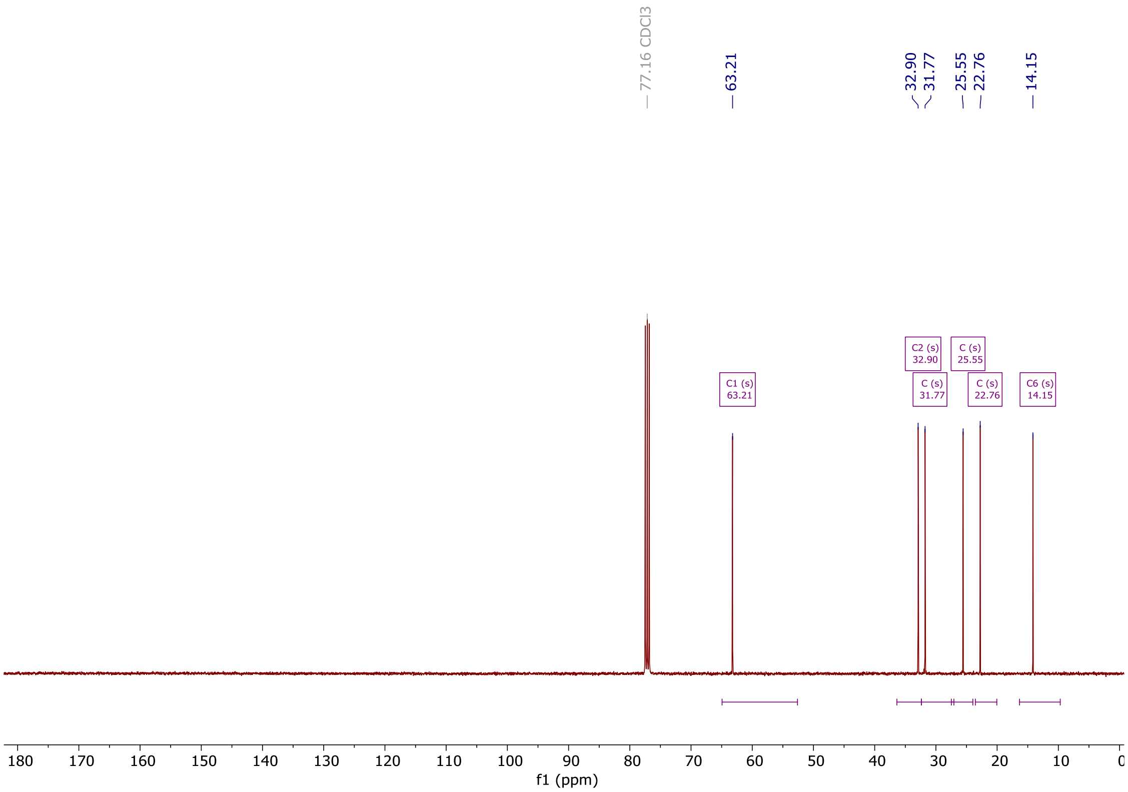
77.16 CDCl3
63.21
32.90
31.77
25.55
22.76
14.15
C1 (s) 63.21
C2 (s) 32.90
C (s) 31.77
C (s) 25.55
C (s) 22.76
C6 (s) 14.15
f1 (ppm)

3-Phenylpropanal oxime **8c**

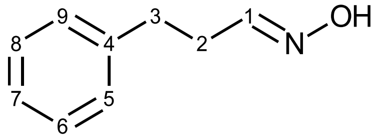

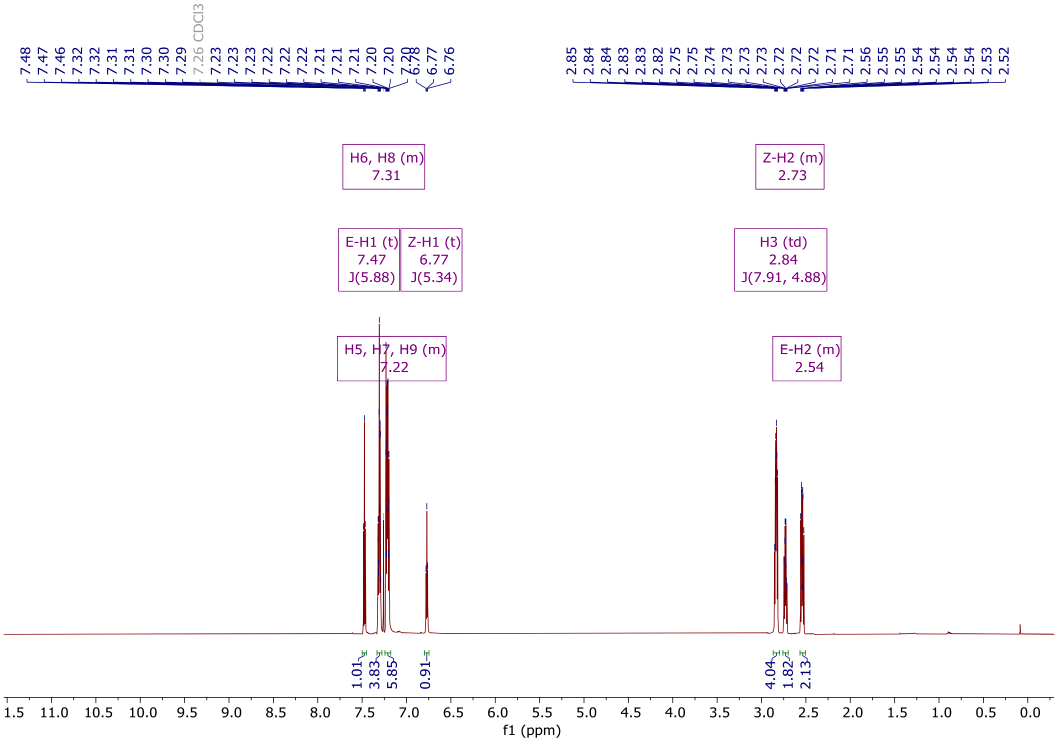

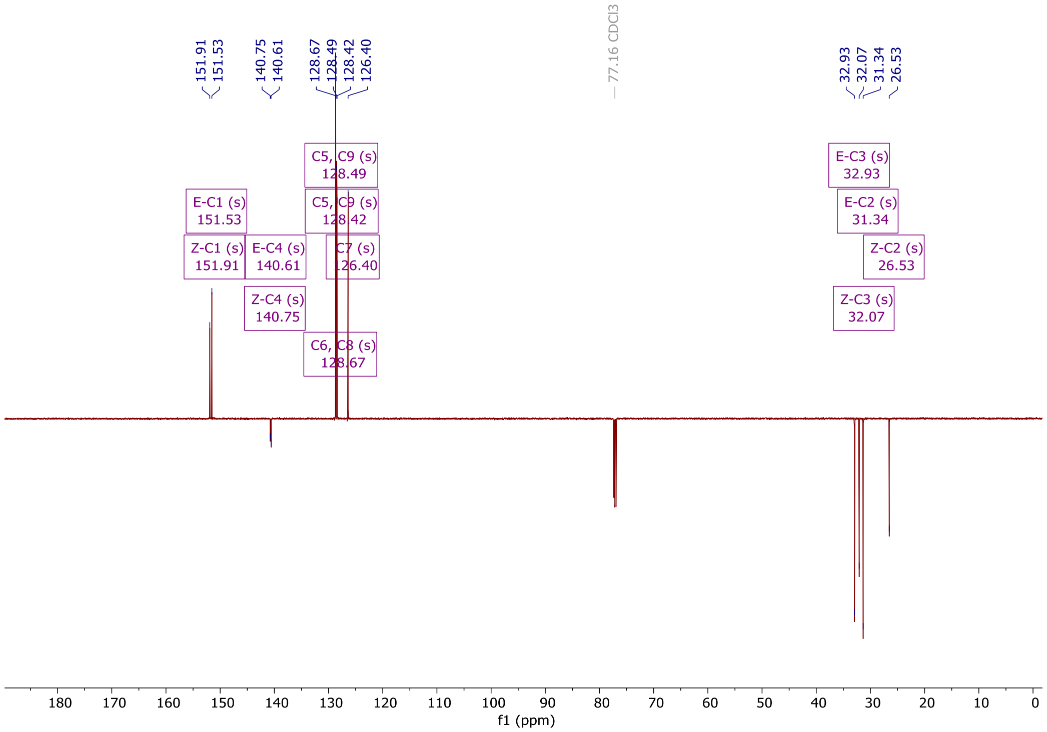

4. References

[1] M. Horvat, G. Fiume, S. Fritsche, M. Winkler, *J. Biotechnol.* **2019**, *304*, 44–51.
[2] M. Winkler, C. K. Winkler, *Monatshefte für Chemie - Chem. Mon.* **2016**, *147*, 575–578.
[3] M. Horvat, S. Fritsche, R. Kourist, M. Winkler, *ChemCatChem* **2019**, *11*, 4171–4181.
[4] Y. Duan, P. Yao, X. Chen, X. Liu, R. Zhang, J. Feng, Q. Wu, D. Zhu, *J. Mol. Catal. B Enzym.* **2015**, *115*, 1–7.
[5] T. Li, J. P. N. Rosazza, *J. Bacteriol.* **1997**, *179*, 3482–3487.
[6] D. Schwendenwein, A. Ressmann, M. Dörr, M. Höhne, U. Bornscheuer, M. Mihovilovic, F. Rudroff, M. Winkler, *Adv. Synth. Catal.* **2019**, adsc.201900155.
[7] J. G. Ling, M. H. Mansor, A. M. Abdul Murad, R. Mohd. Khalid, D. H. X. Quay, M. Winkler, F. D. Abu Bakar, *J. Biotechnol.* **2020**, *307*, 55–62.
[8] M. K. Akhtar, N. J. Turner, P. R. Jones, *Proc. Natl. Acad. Sci.* **2013**, *110*, 87–92.
[9] S. Ebner, M. Horvat, T.-S. Larch, M. Winkler, in *Biotrans 2021*, Graz, Austria, **2021**.
[10] Y. Hu, Z. Zhu, D. Gradischnig, M. Winkler, J. Nielsen, V. Siewers, *Proc. Natl. Acad. Sci.* **2020**, *117*, 22974–22983.
[11] G. G. Gross, *Eur. J. Biochem.* **1972**, *31*, 585–592.
[12] D. Schwendenwein, G. Fiume, H. Weber, F. Rudroff, M. Winkler, *Adv. Synth. Catal.* **2016**, *358*, 3414–3421.
[13] H. Stolterfoht, G. Steinkellner, D. Schwendenwein, T. Pavkov-Keller, K. Gruber, M. Winkler, *Front. Microbiol.* **2018**, *9*, 250.
[14] B. Křístková, N. Kulik, L. Rucká, R. Rädisch, M. Pátek, M. Horvat, M. Winkler, L. Martínková, **2021**.
[15] R. Rädisch, M. Chmátal, L. Rucká, P. Novotný, L. Petrásková, P. Halada, M. Kotik, M. Pátek, L. Martínková, *Int. J. Biol. Macromol.* **2018**, *115*, 746–753.
[16] M. Horvat, V. Weilch, R. Rädisch, S. Hecko, A. Schiefer, F. Rudroff, B. Wilding, N. Klempier, M. Pátek, L. Martínková, M. Winkler, *Catal. Sci. Technol.* **2022**, *12*, 62–66.
[17] M. Horvat, T. S. Larch, F. Rudroff, M. Winkler, *Adv. Synth. Catal.* **2020**, *362*, 4673–4679.
[18] T. V. Hansen, P. Wu, V. V. Fokin, *J. Org. Chem.* **2005**, *70*, 7761–7764.
[19] A. Hinzmann, S. Glinski, M. Worm, H. Gröger, *J. Org. Chem.* **2019**, *84*, 4867–4872.
[20] A. Hinzmann, M. Stricker, H. Gröger, *ACS Sustain. Chem. Eng.* **2020**, *8*, 17088–17096.
